# Construction and analysis of an artificial consortium based on the fast-growing cyanobacterium *Synechococcus elongatus* UTEX 2973 to produce the platform chemical 3-hydroxypropionic acid from CO_2_

**DOI:** 10.1186/s13068-020-01720-0

**Published:** 2020-05-06

**Authors:** Li Zhang, Lei Chen, Jinjin Diao, Xinyu Song, Mengliang Shi, Weiwen Zhang

**Affiliations:** 1 Laboratory of Synthetic Microbiology, School of Chemical Engineering & Technology, Tianjin University, Tianjin, 300072 People’s Republic of China; 2 Frontier Science Center of Synthetic Biology, Key Laboratory of Systems Bioengineering, Ministry of Education of China, Tianjin University, Tianjin, People’s Republic of China; 3 SynBio Research Platform, Collaborative Innovation Center of Chemical Science and Engineering (Tianjin), Tianjin, People’s Republic of China; 4 Center for Biosafety Research and Strategy, Tianjin University, Tianjin, People’s Republic of China

**Keywords:** Cyanobacteria, Artificial consortium, 3-Hydroxypropionic acid, Symbiotic

## Abstract

**Background:**

Cyanobacterial carbohydrates, such as sucrose, have been considered as potential renewable feedstock to support the production of fuels and chemicals. However, the separation and purification processes of these carbohydrates will increase the production cost of chemicals. Co-culture fermentation has been proposed as an efficient and economical way to utilize these cyanobacterial carbohydrates. However, studies on the application of co-culture systems to achieve green biosynthesis of platform chemicals are still rare.

**Results:**

In this study, we successfully achieved one-step conversion of sucrose derived from cyanobacteria to fine chemicals by constructing a microbial consortium consisting of the fast-growing cyanobacterium *Synechococcus elongatus* UTEX 2973 and *Escherichia coli* to sequentially produce sucrose and then the platform chemical 3-hydroxypropionic acid (3-HP) from CO_2_ under photoautotrophic growth conditions. First, efforts were made to overexpress the sucrose permease-coding gene *cscB* under the strong promoter *P*_*cpc560*_ in *S. elongatus* UTEX 2973 for efficient sucrose secretion. Second, the sucrose catabolic pathway and malonyl-CoA-dependent 3-HP biosynthetic pathway were introduced into *E. coli* BL21 (DE3) for heterologous biosynthesis of 3-HP from sucrose. By optimizing the cultivation temperature from 37 to 30 °C, a stable artificial consortium system was constructed with the capability of producing 3-HP at up to 68.29 mg/L directly from CO_2_. In addition, cell growth of *S. elongatus* UTEX 2973 in the consortium was enhanced, probably due to the quick quenching of reactive oxygen species (ROS) in the system by *E. coli*, which in turn improved the photosynthesis of cyanobacteria.

**Conclusion:**

The study demonstrated the feasibility of the one-step conversion of sucrose to fine chemicals using an artificial consortium system. The study also confirmed that heterotrophic bacteria could promote the cell growth of cyanobacteria by relieving oxidative stress in this microbial consortium, which further suggests the potential value of this system for future industrial applications.

## Background

Cyanobacteria are capable of producing organic matter from inorganic carbon (CO_2_) by using solar energy. Due to the challenges associated with global climate change and sustainable energy supply, cyanobacteria have recently attracted significant attention as environmentally friendly and sustainable “microbial cell factories” for the production of biofuels and valuable chemicals directly from CO_2_ [1]. In addition, cyanobacteria have also been considered as a means of producing carbohydrate feedstocks to support industrial fermentative processes [2]. Moreover, it has been reported that several cyanobacterial species are capable of synthesizing and secreting sucrose as an osmolyte under appropriate environmental stimuli, such as osmotic pressure [3], and this production can be sustained over long time periods and at higher levels than that from plant-based feedstocks such as sugarcane and beet [4, 5]. As sucrose is an easily fermentable feedstock for many microorganisms [6, 7], significant efforts have been made to improve the production of extracellular sucrose in cyanobacteria [8]. For example, Du et al. achieved sucrose productivity at 1.43 mg/L/h in wild-type *Synechocystis* sp. PCC 6803 under 600 mM NaCl stress in a bioreactor and doubled the productivity to 3.13 mg/L/h by co-overexpressing key genes related to sucrose synthesis, namely, *sps* (*slr0045*), *spp* (*slr0953*) and *ugp* (*slr0207*), and deleting the glucosylglycerol phosphate synthase gene *ggpS* (*sll1566*) [9]. In another study, Ducat et al. integrated a *cscB* gene encoding sucrose permease from *Escherichia coli* W [10, 11] into the *Synechococcus elongatus* PCC 7942 genome and silenced the carbon competition pathway by knocking out the invertase *inv*A and ADP-glucose pyrophosphorylase *glg*C to achieve sucrose secretion at a rate of 36.1 mg/L/h [12]. Recently, the fast-growing cyanobacterium *Synechococcus elongatus* UTEX 2973 (hereafter *S. elongatus* UTEX 2973) with a growth rate similar to that of yeast was identified [13], and an extracellular sucrose productivity of 35.5 mg/L/h was demonstrated in an engineered *S. elongatus* UTEX 2973 carrying the sucrose transporter *cscB* in a bioreactor experiment [14]. Sucrose productivity was further increased to 79.2 mg/L/h through upregulation of *sps,* which encodes a sucrose-phosphate synthase enzyme, and sucrose synthesis genes in *S. elongatus* UTEX 2973 [15]. In addition to the high rate of sucrose secretion and growth, this strain exhibits high tolerance to high-temperature (41 °C) and high-light (500 μmol photons∙m^−2^∙s^−1^) conditions, suggesting significant advantages for outdoor cultivation in the future [13]. However, as purification of sucrose from culture supernatant is costly and the system is easily contaminated when sucrose is produced at a large scale [16], alternative ways of utilizing sucrose produced by cyanobacteria need to be developed for potential biotechnological applications.

In nature, microorganisms typically live and interact with other microbes by establishing a stable interchange of substances in complex communities [17, 18]. Inspired by the commonly found symbiotic relationships of various microbes in nature, studies have been conducted to simulate symbiotic systems by designing artificial routes for the interchange of substances [19, 20]. Very recently, Ducat et al. constructed a co-culture system with the cyanobacterium *S. elongatus* PCC 7942 and the heterotrophic bacterium *Halomonas boliviensis*, in which the growth of *H. boliviensis* was supported by sucrose produced by *S. elongatus* PCC 7942 [21], and Li et al. constructed a co-culture system consisting of the sucrose secretory cyanobacterium *S. elongatus* PCC 7942 and three different yeasts to mimic lichen and research the interaction between the autotrophic and heterotrophic strains [22]. Although these studies have established new alternatives for the utilization of sucrose derived from cyanobacteria, the use of co-culture systems to achieve one-step conversion of sucrose to fine chemicals is still rare [23]. In addition, compared with axenic cultures of cyanobacteria, autotrophic–heterotrophic symbiotic systems have been found to resist contamination effectively and exhibit good robustness in fluctuating environments [24, 25].

3-Hydroxypropionic acid (3-HP, C_3_H_6_O_3_), as an important platform chemical, is widely used for the production of many chemicals, such as acrylic acid, malonic acid and biodegradable plastic poly-3-hydroxypropionic acid, and can also be used as a food additive or preservative [26]. As chemical synthesis of 3-HP causes severe environmental pollution [27], biosynthesis of 3-HP has attracted significant attention recently. Several 3-HP biosynthetic pathways have been reported, and at least four substrates have been used to produce 3-HP, including β-alanine [28], lactate [29], malonyl-CoA [30] and glycerol [31]. Among these pathways, the malonyl-CoA-dependent pathway, which employs acetyl-CoA carboxylase to convert the precursor acetyl-CoA to malonyl-CoA and malonyl-CoA reductase to convert malonyl-CoA to 3-HP [32, 33], was reported to have some distinct advantages over other pathways, such as a broad feedstock spectrum, thermodynamic feasibility, and redox neutrality [34]. To date, the malonyl-CoA-dependent pathway has been engineered in *E. coli* [35], *Saccharomyces cerevisiae* [36], *Synechocystis* sp. PCC 6803 [37] and *S. elongatus* PCC 7942 [38] for both heterotrophic and photoautotrophic production of 3-HP [35]. However, until now, no study about the biosynthesis of 3-HP by a co-culture system has been reported.

**Fig. 1:**
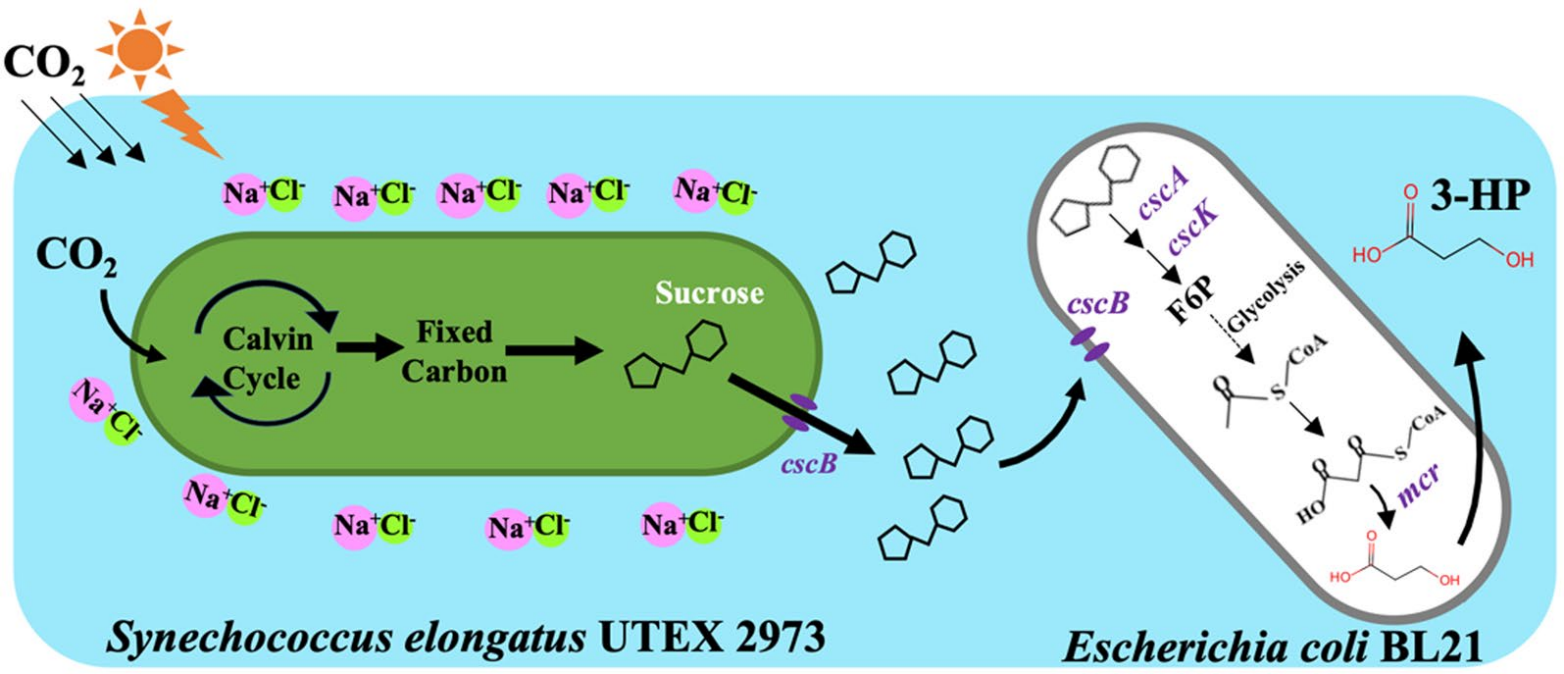
Schematic diagram of the artificial consortium system. The engineered *S. elongatus* UTEX 2973 secreted sucrose under osmotic stress to support *E. coli* growth and synthesis of 3-HP under photoautotrophic growth conditions (F6P, fructose 6-phosphate; 3-HP, 3-hydroxypropionic acid)

In this study, we reported the construction of an artificial consortium system consisting of the fast-growing cyanobacterium *S. elongatus* UTEX 2973 and an engineered *E. coli* BL21(DE3) to produce 3-HP under photoautotrophic conditions. In the consortium system, *E. coli* BL21(DE3) was genetically modified to synthesize 3-HP using sucrose produced by the engineered *S. elongatus* UTEX 2973 (Fig. 1). With the application of this co-culture system, the final yield of 3-HP was approximately 68.29 mg/L, which is comparable to that obtained in *E. coli* when only malonyl-CoA reductase was overexpressed [30]. In addition to the relationship where *S. elongatus* provides sucrose as a carbon source for growth and 3-HP production in *E. coli*, the study also found that increased expression of reactive oxygen species (ROS)-quenching genes in *E. coli* may promote cyanobacterial growth by relieving oxidative stress in the environment.

## Results

### Growth of *S. elongatus cscB*^+^ 2973 and sucrose secretion

**Fig. 2:**
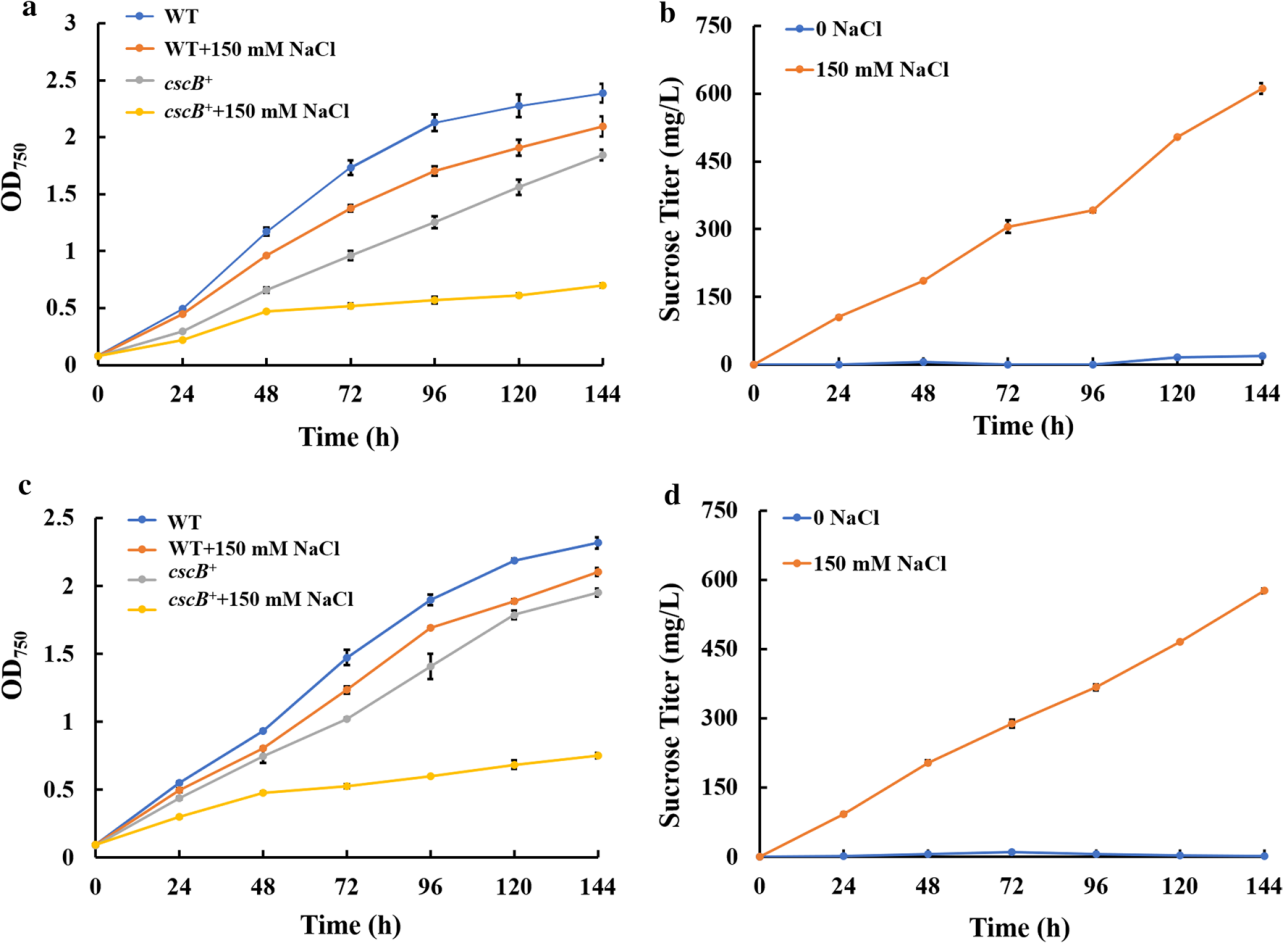
Effects of NaCl on the growth and sucrose yield of *S. elongatus cscB*^+^. Growth of strain *S. elongatus cscB*^+^ in BG-11 medium (**a**) and CoBG-11 medium (**c**). Sucrose yield of strain *S. elongatus cscB*^+^ in BG-11 medium (**b**) and CoBG-11 medium (**d**). The error bars represent the calculated standard deviation of the measurements of three biological replicates

*Synechococcus elongatus* UTEX 2973 was engineered to secrete sucrose by expressing the sucrose permease-encoding gene *cscB* (*ECW_m2594*) under the strong promoter *P*_*cpc560*_. Sucrose secretion from *S. elongatus cscB*^+^ 2973 is mainly dependent upon the pH and NaCl concentration of the medium, and an alkaline environment was previously reported to be beneficial for sucrose secretion from cyanobacterial cells [39]. We used an alkaline environment (pH ≈ 8.3) with 150 mM NaCl (37 °C) to ensure the production and secretion of sucrose from *S. elongatus cscB*^+^ 2973 [14, 22]. The sucrose yield and the growth of *S. elongatus cscB*^+^ 2973 in different culture media are compared in Fig. 2. The results showed that no sucrose was produced from *S. elongatus cscB*^+^ 2973 cells without NaCl in the culture medium. However, sustainable production and secretion of sucrose could be observed for 6 days when 150 mM NaCl was added, and titers of 612.0 mg/L and 576.5 mg/L sucrose were achieved when *S. elongatus cscB*^+^ 2973 was grown in BG-11 and CoBG-11, respectively. To maintain the growth of *E. coli* in co-culture medium, the effect of different salt concentrations on cell growth was also examined (Additional file 1: Fig. S1), and the results showed that *E. coli* was able to grow normally under the tested range of salt concentrations. In this study, a sucrose titer of 576.5–612.0 mg/L (4.00–4.25 mg/L/h) was achieved by *S. elongatus cscB*^+^ 2973 cells over 6 days, which is comparable to the levels observed in similar studies conducted previously (Table 1). For example, although no CO_2_ aeration occurred during *S. elongatus* cultivation, sucrose secretion in this study was still higher than the 2.2 mg/L/h value reported in a previous study [22].

**Table 1:** Sucrose yield in different cyanobacteria strains under co-culture conditions

Host strains	Genotype	Cultural condition	Cultural medium	Titer (mg/L)	Productivity (mg/L/h)	Source of value
*Synechococcus elongatus* PCC 7942	NS3::*P*_*lac*_*-cscB*-Cm^r^	30 °C or 37 °C ^a^, 2 % CO_2_, 65 μE m^-2^ s^-1^ light, 150 mM NaCl for 2 days (baffled flasks)	^CoY^BG-11 or ^CoB^BG-11^b^	156–625	3.25–13.02	[25]
	NS3::*P*_*lac*_*-cscB*-Cm^r^	28 °C ^c^, 1% CO_2_, 65 μmol m^-2^ s^-1^ with 16:8 h light/dark cycle 100 mM NaCl For 4 days(baffled flasks)	BG-11 supplemented with 1 g/L HEPES (pH 8.9)	~210	~2.2	[22]
	NS3::*P*_*lac*_*-cscB*-Cm^r^	35 °C, 2 % CO_2_, 65 μE m^-2^ s^-1^ light, 140 mM NaCl for 3 days(bioreactor)	M1^d^	~250	~3.47	[21]
*Synechococcus elongatus* UTEX 2973	pJA-*cscB*	37 °C, 100 μE m^−2^ s^−1^ light, 150 mM NaCl for 3 days(100-ml round flask)	CoBG-11	295.5	~4.11	This study
	pJA-*cscB*	30 °C, 100 μE m^−2^ s^−1^ light, 150 mM NaCl for 3 days(100-ml round flask)	CoBG-11	288	~4.00	This study

^a^In co-culture system, 37°C for *E. coli* and *B. subtilis*, and 30°C for *S. cerevisiae*

^bCoB^BG-11 consists of BG-11 supplemented with 106 mM NaCl, 4 mM NH_4_Cl and 25 mM HEPPSO, pH-8.3 KOH. ^CoY^BG-11 consists of BG-11 supplemented with 0.36 g/L yeast nitrogen base without amino acids (Sigma Aldrich), 106 mM NaCl, 25 mM HEPPSO, pH 8.3-KOH and 1 mM KPO_3_

^c^In co-culture system, 28°C for three yeast strains

^d^M1 consists BG-11 medium was additionally supplemented with 15 mM NaNO_3_, 4.5 mM K_2_HPO_4_ (phosphate buffering), 1.5 mM MgSO_4_, 5 mM Na_2_SO_4_, 30 μM FeCl_3_, 30 μM Na_2_MoO_4_, and 1x additional trace metals before the addition of KOH to pH 8.3

### Growth of an engineered *E. coli* mutant in co-culture medium

**Fig. 3:**
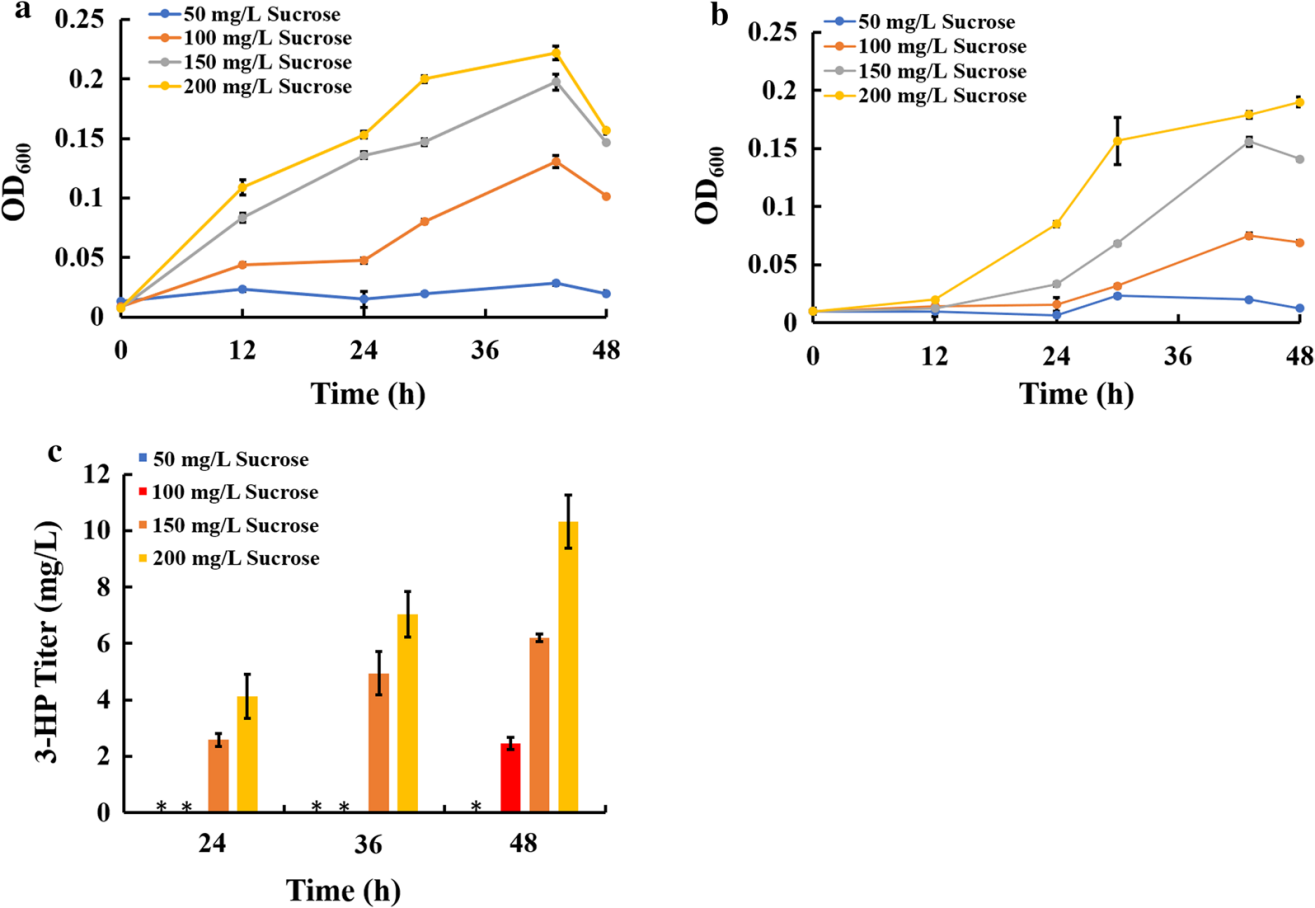
Growth and 3-HP production of pure *E. coli* ABKm culture under different sucrose concentrations. **a** Cultivated in M9 medium; **b** cultivated in CoBG-11 medium. The error bars represent the calculated standard deviation of the measurements of three biological replicates. **c** 3-HP production in the *E. coli* ABKm strain under different initial sucrose concentrations in co-culture medium (“_*_” indicates that no detectable amount was observed). The error bars represent the calculated standard deviation of the measurements of three biological replicates

To ensure that *E. coli* BL21 utilizes sucrose as the sole carbon source, we cloned and expressed the essential genes for sucrose metabolism, namely, *cscB* (*ECW_m2594*), *cscK* (*ECW_m2595*) and *cscA* (*ECW_m2596*), into *E. coli* BL21 to generate an engineered strain, *E. coli* cscN. In addition, to synthesize 3-HP, the malonyl-CoA reductase-coding gene *mcr* (*Caur_2614*) was introduced into *E. coli* cscN, resulting in the engineered strain *E. coli* ABKm. A growth comparison of these two strains is shown in Additional file 2: Fig. S2. As shown, under the same conditions, the final cell density of cscN without the *mcr* gene was slightly increased. In a previous study, an artificial consortium was constructed by inoculating a heterotrophic bacterium into a *S. elongatus* PCC 7942 culture with OD_750_ = 0.5 [25]. In our study, the sucrose yield of *S. elongatus cscB*^+^ 2973 was ~ 200 mg/L when the cells reached OD_750_ = 0.5. Therefore, we selected four concentrations, namely, 50, 100, 150, and 200 mg/L, to examine whether *E. coli* ABKm could be stably maintained in the system using these levels of sucrose as the sole carbon source in M9 and CoBG-11 media (Fig. 3a, b). The growth of *E. coli* ABKm could be detected under 100, 150, and 200 mg/L sucrose. A previous study showed that the *E. coli* Δ*cscR* strain required a minimal sucrose concentration of 1.2 g/L for growth [25], which is much higher than our result for strain ABKm, suggesting that after expression of *cscA*, *cscB* and *cscK*, the efficiency of sucrose utilization might have improved in strain ABKm [25, 40]. To demonstrate that this effect was not caused by a strain-specific difference, the sucrose utilization pathway was also engineered into *E. coli* MG1655 and BW25113, and a similar result was observed (data not shown). Additionally, we also determined the 3-HP yield in strain ABKm with the different concentrations of sucrose mentioned above (i.e., 50–200 mg/L) in CoBG-11, and the results showed that strain ABKm was able to produce 3-HP under all the concentrations except 50 mg/L sucrose (Fig. 3c).

### Establishing a stable artificial consortium to produce 3-HP

**Fig. 4:**
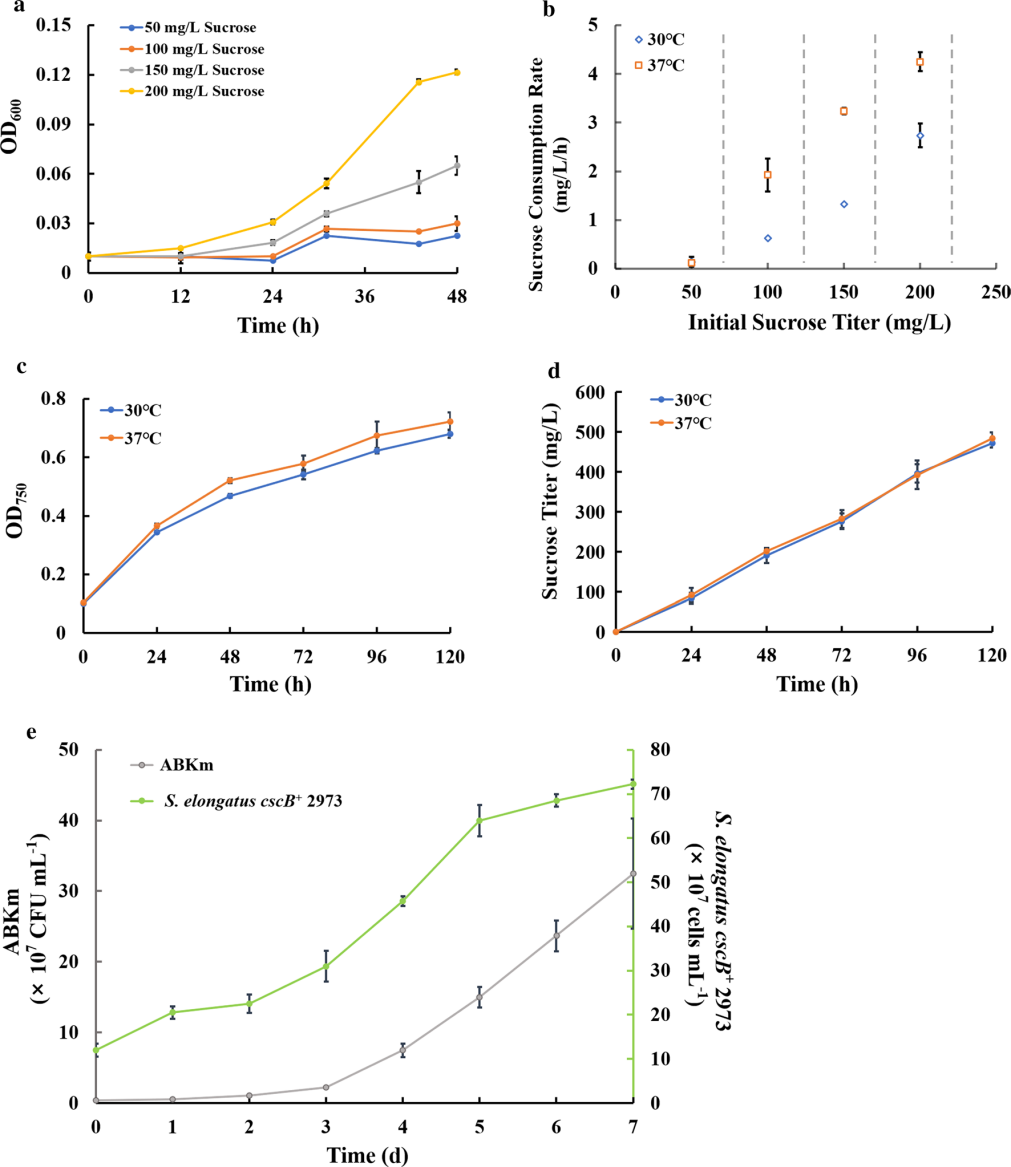
Construction of an artificial consortium system with engineered *S. elongatus* UTEX 2973 and *E. coli* BL21. **a** Growth of pure ABKm culture in CoBG-11 medium with different concentrations of sucrose at 30 °C. **b** Sucrose utilization rate of pure ABKm culture. **c** Growth of pure *S. elongatus* UTEX 2973 culture at 30 °C and 37 °C. **d** Sucrose yield of engineered *S. elongatus cscB*^+^. **e** Growth of two strains in the artificial consortium system at 30 °C. The error bars represent the calculated standard deviation of the measurements of three biological replicates

Since the optimal growth temperature for both *E. coli* and *S. elongatus* UTEX 2973 is 37 °C, we initially set this as the incubation temperature for the co-culture system. However, the analysis showed that *E. coli* strain ABKm grew poorly after 1–2 days in this system compared with the growth observed in a previous study [25] (Additional file 3: Fig. S3). According to the data (Fig. 2d), at 37 °C, *S. elongatus cscB*^+^ 2973 cells produce a sufficient amount of sucrose, which prompted us to hypothesize that the rapid cell growth of *E. coli* and utilization of sucrose destroy the balance of the two species in this system. To confirm this hypothesis, we determined the rates of sucrose secretion and sucrose utilization in *S. elongatus* UTEX 2973 and *E. coli*, respectively. The results showed that the sucrose utilization rate of *E. coli* strain ABKm increased gradually with increasing initial sucrose concentration, reaching ~ 4.20 mg/L/h at an initial sucrose concentration of 200 mg/L with growth at 37 °C for 48 h (Fig. 4b). Although the sucrose secretion rate of *S. elongatus cscB*^+^ could reach ~ 4.11 mg/L/h, we speculated that with the accumulation of *E. coli* biomass, the sucrose consumption rate of ABKm could be faster than the sucrose secretion rate of *S. elongatus cscB*^+^ at 37 °C. Therefore, the “production–consumption” balance was disrupted, leading to collapse of the consortium. These results led us to adjust the cultivation temperature from 37 °C to 30 °C, aiming to slow down the consumption of *E. coli* and achieve balanced growth of *S. elongatus* UTEX 2973 and *E. coli* in the system. The growth of strain ABKm at 30 °C was then observed (Fig. 4a), and the sucrose utilization rate of this strain was determined to be ~ 2.00 mg/L/h at 30 °C at 48 h (Fig. 4b). Interestingly, there was no significant difference between 37 °C and 30 °C in terms of cell growth and sucrose production of the *S. elongatus cscB*^+^ 2973 strain (Fig. 4c, d). As a result, the artificial consortium with *S. elongatus cscB*^+^ 2973 and *E. coli* strain ABKm was successfully constructed and could be maintained stably for at least 7 days at 30 °C (Fig. 4e).

**Fig. 5:**
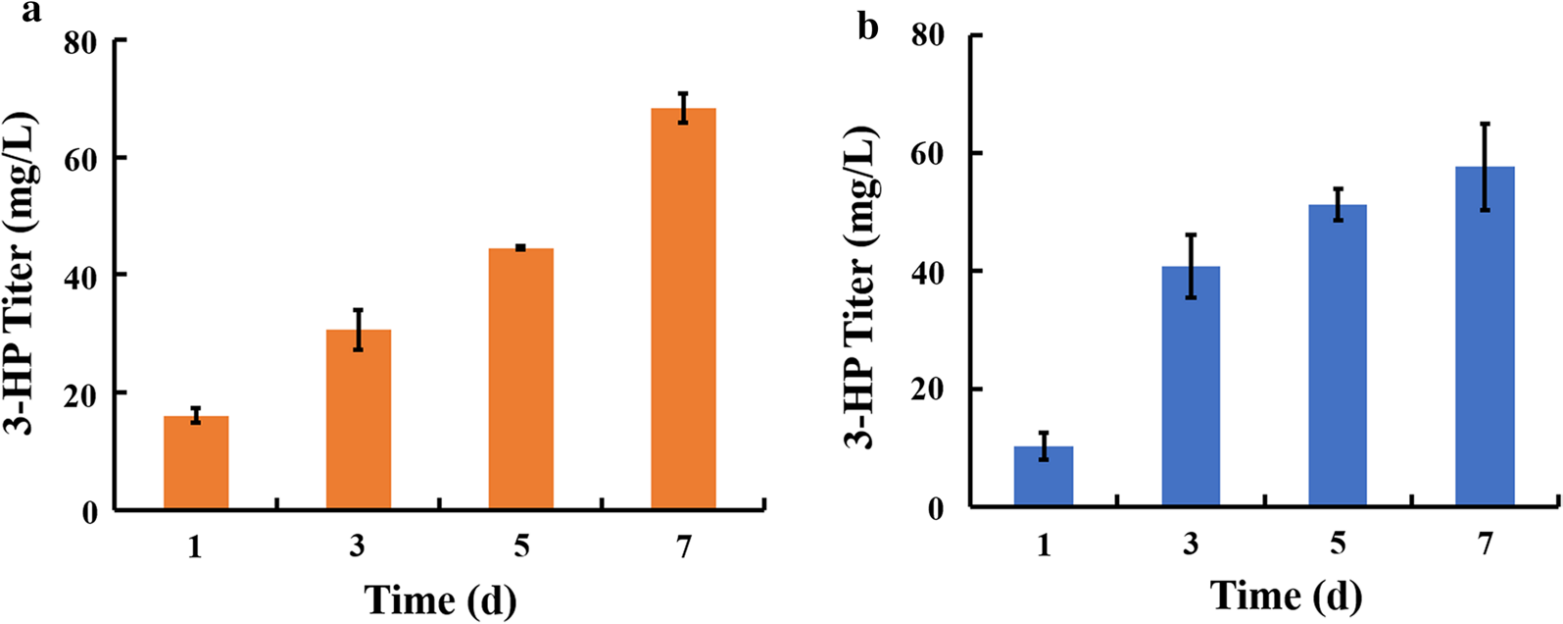
3-HP yield under different conditions. **a** 3-HP yield in an artificial consortium system. **b** 3-HP production in strain ABKm under continuous supplementation with sucrose according to the calculated sucrose secretion rate of *S. elongatus cscB*^+^. The error bars represent the calculated standard deviation of the measurements of three biological replicates

**Fig. 6:**
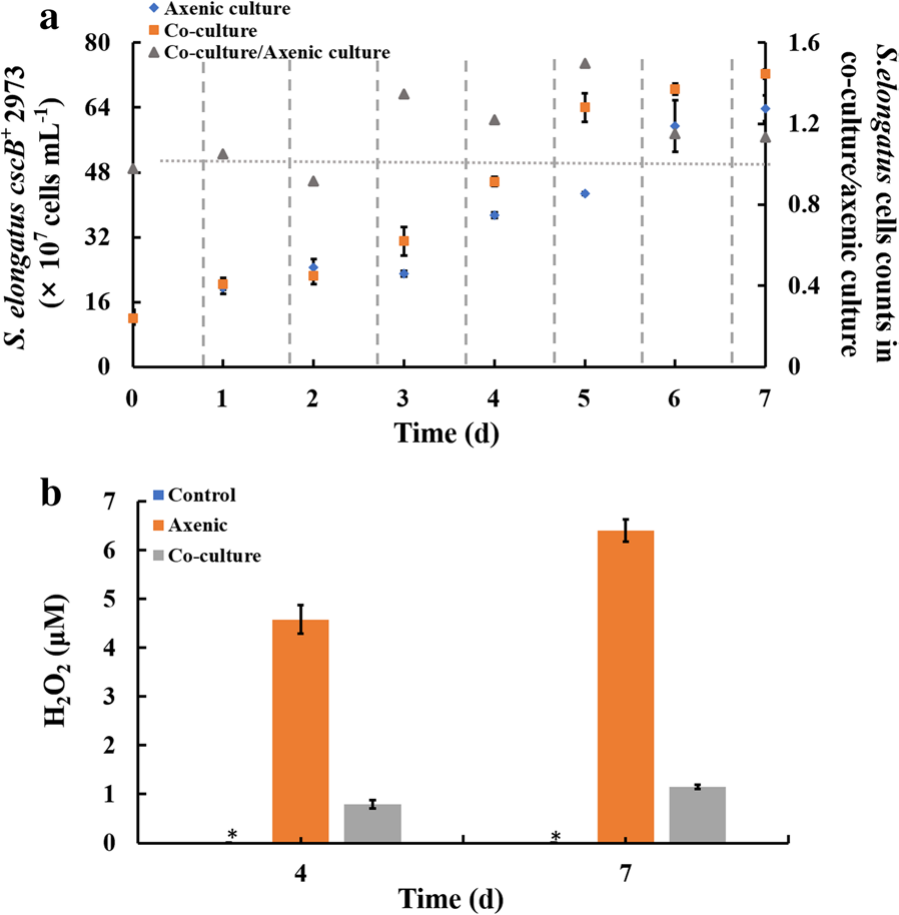
Growth of cyanobacteria and H_2_O_2_ content in different culture conditions. **a** Difference in the growth of *S. elongatus cscB*^+^ in the artificial consortium and under axenic conditions. The blue rhombus represents the cell number in the axenic consortium, the orange rectangle represents the cell number in the artificial consortium, and the gray triangle represents the ratio between the two conditions. **b** H_2_O_2_ content in blank CoBG-11 (control), axenic culture and co-culture systems under the same conditions (30 °C, 100 μE m^−2^s^−1^ light, 150 mM NaCl; “_*_” indicates that no detectable amount was observed under the conditions). The error bars represent the calculated standard deviation of the measurements of three biological replicates

To evaluate the production capacity of the artificial consortium, the 3-HP yield of *E. coli* strain ABKm was analyzed. As shown in Fig. 5a, 3-HP production reached ~ 68.29 mg/L in 7 days. In parallel, we also determined 3-HP production in *E. coli* strain ABKm under pure culture conditions with continuous supplementation of sucrose according to the calculated sucrose secretion rate of *S. elongatus cscB*^+^ 2973 (Fig. 5b), and the results showed that the 3-HP yield under pure conditions was at the same level. In addition, we also observed that *S. elongatus cscB*^+^ 2973 cultivated in the consortium grew better than the cells cultivated in pure culture conditions (Fig. 6a), consistent with previous findings [25]. In addition, the results showed that almost no free sucrose could be detected in the co-culture medium, suggesting that sucrose produced by the cyanobacterium was completely consumed by the *E. coli* ABKm strain to support cell growth and accumulate the desired product (Additional file 4: Fig. S4) [25].

### Effect of oxidative stress on cyanobacteria in an artificial consortium system

ROS are common byproducts of aerobic metabolic processes, such as photoreactions and respiration, in oxygenic photosynthetic organisms [41], and ROS accumulation could cause oxidative damage to cyanobacterial cells. In addition, previous studies have found that organic buffers in culture media may also contribute to the generation of H_2_O_2_ [42]. For example, 1 ~ 10 mM 4-(2-hydroxyethyl)1-piperazineethanesulfonic acid (HEPES) in culture medium could produce enough H_2_O_2_ to kill *Prochlorococcus* [43]. Since there was also organic buffer (TES) used to maintain pH in our study, to clarify whether this organic buffer generates H_2_O_2_, we determined the titer of H_2_O_2_ in blank CoBG-11 under the same culture conditions. The results showed that no H_2_O_2_ was detected in blank culture medium, suggesting that the H_2_O_2_ in culture medium was mostly synthesized from living cells. Next, we examined the impact of *E. coli* co-cultivation on the H_2_O_2_ level, and the results showed that the H_2_O_2_ content was significantly reduced when the heterotrophic partner of *E. coli* was included in the system (Fig. 6b), which is consistent with a previous study [22].

**Fig. 7:**
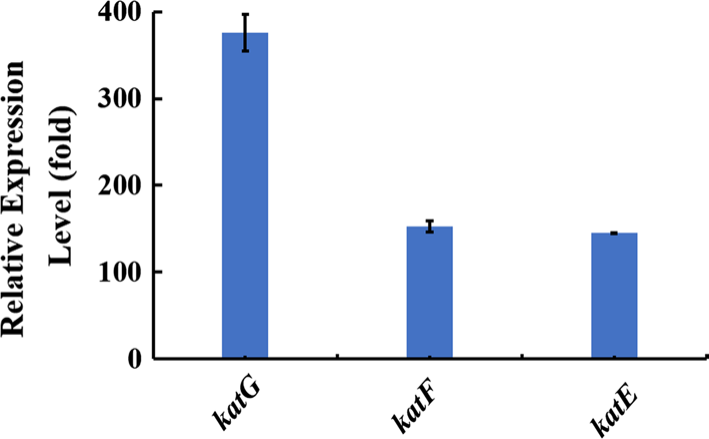
Expression level analysis of genes involved in ROS quenching. Gene expression analysis of *katG, katF* and *katE* in the ABKm strain

To further understand this phenomenon at the molecular level, the expression levels of several H_2_O_2_-quenching genes in the *E. coli* ABKm strain under pure and co-culture conditions were comparatively analyzed by qRT-PCR (Additional file 5: Table S1). It is well known that *E. coli* contains three types of catalases: hydroperoxidase I (HPI) (*katG*), hydroperoxidase II (HPII) (*katF*), and hydroperoxidase III (HPIII) (*katE*) [44–46]. In addition, the synthesis of HPII often increases markedly when cells enter the stationary phase [47, 48]. The transcriptional expression of these three genes was determined. As shown in Fig. 7, the relative expression levels of *katG*, *katF* and *katE* in the *E. coli* ABKm strain were dramatically upregulated under co-culture conditions compared with those in CoBG-11 under continuous supplementation with sucrose according to the calculated sucrose secretion rate of *S. elongatus cscB*^+^ 2973, suggesting that *E. coli* might be able to remove ROS when co-cultivated with cyanobacterial partners and thus possibly alleviate the overall oxidative stress in the consortium system.

## Discussion

Photosynthetic cyanobacteria have been considered an important alternative for providing sustainable feedstock, and cyanobacterial carbohydrates have been considered a potential renewable feedstock to support the production of fuels and chemicals. However, since axenic cultures of cyanobacteria are vulnerable to contamination [49] and it is expensive to separate and purify these products from culture medium [50], their application is limited. To address these issues, the use of synthetic consortia of cyanobacteria paired with specific heterotrophic partners has been proposed. The fast-growing *S. elongatus* UTEX 2973 strain reported recently exhibits high tolerance to high-temperature and high-light conditions [13]. In this study, we reported the construction of an artificial co-culture system utilizing *S. elongatus* UTEX 2973 and *E. coli* BL21 to produce the heterologous chemical 3-HP directly from CO_2_. In addition, analysis of the mechanism underlying the co-culture systems also provided a fundamental basis for further optimization of artificial consortium systems via metabolic engineering.

Sucrose is a commonly used carbon source for industrial microbial fermentation [51, 52]. In this study, we transformed the sucrose transporter *cscB* into the fast-growing cyanobacterium *S. elongatus* UTEX 2973 and obtained the sucrose-secreting strain *S. elongatus cscB*^+^ 2973 with a productivity of 612.0 mg/L BG-11 in 6 days. To construct an artificial consortium using sucrose as the sole carbon source, the sucrose utilization ability of *E. coli* was also improved. It was previously shown that sucrose utilization could be endowed in the *Pseudomonas putida cscAB* strain by introducing the *cscA* and *cscB* genes from *E. coli* W [23]. To achieve the growth of *E. coli* BL21 in the designed co-culture system, genes related to sucrose utilization, namely, *cscA*, *cscB* and *cscK*, were introduced [53]. The results showed that this engineering strategy enabled *E. coli* strain ABKm to grow on sucrose as the sole carbon source (Fig. 3). A previous study reported that *E. coli ΔcscR* strain required a minimal sucrose concentration of 1.2 g/L for growth, which is much higher than the concentration required by ABKm here, suggesting that this engineering strategy significantly improved the efficiency of sucrose utilization [40].

It was previously reported that ROS accumulation in the culture medium severely inhibited axenic growth of cyanobacteria; however, this effect was efficiently alleviated through co‑culture with *Rhodotorula glutinis* [22]. We also observed improved growth of *S. elongatus* UTEX 2973 in the co-culture system (Fig. 6a). The H_2_O_2_ content in the consortium system was found to be lower than that in the axenic culture, suggesting that *E. coli* might contribute to the alleviation of ROS stress and thus promote the growth of cyanobacteria in the artificial consortium, which was further confirmed by the upregulation of ROS quenching-related genes in *E. coli* strain ABKm (Fig. 7). In addition, to confirm our hypothesis, we also tested the influence of H_2_O_2_ on the cell growth of axenic cyanobacterial cultures, and the results showed that the addition of 10 μM H_2_O_2_ significantly decreased cell growth (Additional file 6: Fig. S5). The upregulation of ROS quenching capability could be important, as ROS may deleteriously affect cellular metabolic processes, such as nutrient bioavailability, photosynthesis and carbon flux, in plants [54]. With the decreased H_2_O_2_ content and increased cell growth of *S. elongatus* UTEX 2973, we also evaluated the expression levels of several genes involved in photosynthesis, including the carbon dioxide-concentrating mechanism protein-coding gene *ccmM* [55], ribulose-1,5-bisphosphate carboxylase/oxygenase (RuBisCO)-coding gene *rbcL* [56], PSII-related subunit-coding genes *cp43* and *cp47*, PSI essential subunits of reaction center-coding genes *psaA* and *psaB* [57], and chlorophyll a synthesis-related genes *chlaA* and *pcrA* [58] (Additional file 5: Table S1); as expected, all these genes were upregulated (Additional file 7: Fig. S6). Several studies have also reported that heterotrophic partners can provide the necessary inorganic carbon by decomposing organic matter and growth factors such as vitamin B_12_ to cyanobacteria in the natural and artificial consortium [18, 59, 60], which we will also analyze in future work. Further analysis of the interactions in the consortium could provide the necessary theoretical basis for the potential application of artificial co-culture systems in many areas, such as controlling blooms [61], degradation of pollutants [62–64], and soil remediation [65–67]. For example, Fedeson et al. created an artificial consortium composed of two bacterial species (*S. elongatus* PCC 7942 and *P. putida*) that enables the degradation of the industrially produced environmental pollutant 2,4-DNT while simultaneously producing polyhydroxyalkanoates (PHA) bioplastic [68].

Our study also provided some insights into the potential relationships between the two species. The first layer of interaction is that the cyanobacterium provides the carbon source for the *E. coli* strain, and the engineered *E. coli* strain consumes this carbon source to grow and produce fine chemicals. Interestingly, we also found that the cell growth of the cyanobacterium was somewhat improved in the co-culture system. By taking a close look at the changes in the factors involved in determining cell growth, we found that *E. coli* may help quench ROS, which in turn promotes the growth of cyanobacteria, which is considered the second layer of interaction between these two species. Collectively, we concluded that the relationship between these two species in the co-culture system is mutualistic.

Although several previous studies have demonstrated promising characteristics of co-culture systems [21, 25], our work showed their new application: first, the final product was different. Unlike polyhydroxybutyrate (PHB), 3-HP is a heterologous compound, and our study provides proof of the concept that the production of heterologous fine chemicals can be achieved in co-culture systems by rational design. Second, *S. elongatus* UTEX 2973 exhibits high tolerance to high-light conditions compared with other model cyanobacterial species, such as *S. elongatus* PCC 7942, which means that the robustness of our system may be improved. Furthermore, compared with traditional “two-stage” fermentation, this “one-step” strategy also has several unique advantages. Besides the issue of bacterial contamination as the excess sugar in the system is consumed by heterotrophic partners [21], the “two-stage” culture strategy typically requires two steps to obtain the chemical production: obtaining the supernatant of the cyanobacterial, and then inoculating the *E. coli* strain to achieve cell growth and chemical production, which can greatly increase the production cost.

Although the artificial consortium system was successfully established and the desired product was obtained in this study, there remain many aspects that need to be improved in the future, such as the yield of 3-HP. To increase the yield of the target product, on the one hand, we could enhance the supply of the carbon source. In addition to silencing the competing consumption pathway [12], Qiao et al. reported that sucrose yield in *S. elongatus* PCC 7942 can be enhanced from 6.5 to 8.0 mg/L/h by overexpressing sucrose-phosphate synthase (*sps*) and glucose-1-phosphate adenylyltransferase (*glgC*) at the same time [69]. The research also suggested that glycogen could serve as a supporting rather than a competitive carbon pool for sucrose synthesis. In addition, Weiss et al. used alginate to encapsulate *S. elongatus* and enhanced the sucrose yield rates ~ twofold within 66 h [21]. On the other hand, heterotrophic partners also need to be improved. Chelladurai et al. developed a variety of recombinant *E. coli* strains by expressing the heterologous gene *mcr* and overexpressing the endogenous acetyl-CoA carboxylase and biotinidase-encoding genes *accADBCb* and nicotinamide nucleotide transhydrogenase-encoding gene *pntAB*, which converts NADH to NADPH in *E. coli*. In addition, several deletion mutations in phosphotransacetylase (*pta*) acetate kinase (*ackA*) and lactate dehydrogenase (*ldhA*) or the α-ketoglutarate dehydrogenase complex (*sucAB*) were carried out with the recombinant strains. The final 3-HP titer was enhanced approximately threefold from 0.71 to 2.14 mM [30]. Moreover, Cheng et al. reported the overexpression of heterogeneous acetyl-CoA carboxylase (from *Corynebacterium glutamicum*) and codon-optimized *mcr* in *E. coli* BL21; three types of modified *E. coli* strains with different host–vector systems were constructed and investigated, and the results showed that the combination of *E. coli* BL21 and pET28a was the most efficient host–vector system for 3-HP production. The concentration of 3-HP was enhanced from 0.68 g/L to 1.80 g/L in shake flask cultivation [70]. These studies provide valuable guidance for further metabolic engineering in *E. coli*.

## Conclusion

With defined composition and controllable functions, synthetic consortia hold great promise for diverse value-added production, bioenergy and environmental applications. In this study, we demonstrated the feasibility of constructing an artificial consortium to achieve the one-step conversion of sucrose to the platform chemical 3-HP directly from CO_2_. With the application of this co-culture system, the final production of 3-HP was approximately 68.29 mg/L, which is comparable to that in *E. coli* when only malonyl-CoA reductase was overexpressed. Meanwhile, this study also confirmed that in this microbial consortium, heterotrophic bacteria could promote the cell growth of cyanobacteria by relieving oxidative stress, which further demonstrates the potential value of this system for the green biosynthesis of chemicals in the future.

## Materials and methods

### Strains, plasmid construction and culture conditions

*Synechococcus elongatus* UTEX 2973 and *E. coli* BL21(DE3) were engineered and applied to the construction of the artificial consortium system. The essential genes for sucrose metabolism, namely, the permease-coding gene *cscB*, invertase-coding gene *cscA* and fructokinase-coding gene *cscK*, were derived from *E. coli* W [53], while the malonyl-CoA reductase gene *mcr* was from *Chloroflexus aurantiacus* [71]. The super-strong promoter *P*_*cpc560*_ was used to direct gene expression in *S. elongatus* UTEX 2973 [72]. All plasmids were prepared in *E. coli* DH5α. The sucrose hydrolysis system was integrated into pET-30a in *E. coli* with kanamycin resistance, and *mcr* was integrated into pACYC184 under the constitutive promoter *P*_*J23100*_ in *E. coli* with spectinomycin resistance.

*Synechococcus elongatus* UTEX 2973 and the resulting engineered strains were cultivated in BG-11 medium (pH 7.5) under a light intensity of approximately 100 μmol photons m^−2^s^−1^ in an illuminating shaking incubator (HNYC-202T, Honour, Tianjin, China) at 130 rpm and 37 °C or on BG-11 agar plates in an incubator (SPX-250B-G, Boxun, Shanghai, China) [73]. To maintain the stable phenotype of sucrose secretion, appropriate antibiotics were added when necessary. *E. coli* strains were grown on LB medium or agar plates with appropriate antibiotics added to maintain plasmids at 37 °C in a shaking incubator (HNY-100B, Honour, Tianjin, China) at 200 rpm or in an incubator, respectively. All strains used in this study are listed in Table 2.

**Table 2:** Plasmids and strains used in this study

Plasmids/strains	Genotype	Source
*E. coli* strains		
DH5α	F^–^ *endA1* *glnV44* *thi-1* *recA1* *relA1* *gyrA96* *deoR* *nupG* *purB20* φ80d*lacZ*ΔM15 Δ(*lacZYA-argF*)U169, hsdR17(*r*_*K*_^–^*m*_*K*_^+^), λ^–^	TransGen Biotech
HB101	*supE*44, Δ(*mcrC-mrr*), *recA*13, *ara*-14, *proA*2, *lacY*1, *galK*2, *rpsL*20, *xyl*-5,*mtl*-1, *leuB*6, *thi-*1	Takara Bio
BL21(DE3)	F^–^ *ompT* *gal* *dcm* *lon* *hsdS*_*B*_(*r*_*B*_^–^*m*_*B*_^–^) λ(DE3)	TransGen Biotech
cscN	BL21(DE3)/pET30a	
ABKm	BL21(DE3)/pACYC184/pET30a	This study
Plasmids		
pACYC184	P_*J23100*_-mcr-T_*7*_; *spe*^*R*^	This study
pET30a	*csc*A, *csc*B, *csc*K; *kan*^*R*^	This study
pJA	P_*cpc560*_-*csc*B-T_*rbcL*_; *spe*^*R*^, *kan*^*R*^	This study
Cyanobacteria strains		
WT	Wild-type *Synechococcus elongatus* UTEX 2973	
*cscB*^+^ 2973	WT/pJA	This study

### Conjugation of *S. elongatus* UTEX 2973

Constructs were delivered into *S. elongatus* UTEX 2973 through conjugation [74]. *E. coli* HB101 harboring pRL443 and pRL623 (named “helper”) and *E. coli* DH5α harboring the plasmid with the target gene were cultivated overnight and then transferred separately into fresh liquid LB medium with the appropriate antibiotics at a 1:50 ratio. When both strains grew to exponential phase (OD_600_ = 0.3–0.5), 10 mL of the cells of each strain was collected by centrifugation and washed with fresh LB medium three times to remove all the antibiotics. Then, 0.1 mL of fresh LB was used to resuspend each strain, and the cells were mixed together and incubated at 37 °C for 30 min. During this time, 10 mL of *S. elongatus* UTEX 2973 cells at exponential phase (OD_750_ ≈ 1) was collected by centrifugation and resuspended in 0.2 mL of fresh BG-11 medium. *S. elongatus* UTEX 2973 cells were mixed with the *E. coli* mixture mentioned above and incubated at 37 °C under light for 30 min. Then, the mixture was spread on BG-11 agar plates, which were then covered by sterile cellulose filters (0.45 μm pore size). The plates were incubated under light at an intensity of approximately 100 μmol photons m^−2^s^−1^ for 24 h, and then, the cellulose filters were transferred onto new BG-11 agar plates with appropriate antibiotics [75].

### Construction of the artificial consortium system

Co-culture medium (named CoBG-11) was designed based on BG-11 medium and optimized for *E. coli* growth by supplementing with 150 mM NaCl, 4 mM NH_4_Cl and 3 g/L 2-[[1,3-dihydroxy-2-(hydroxymethyl) propan-2-yl] amino] ethanesulfonic acid (TES). The pH value was adjusted with NaOH to 8.3. NaCl and NH_4_Cl were used to maintain the cell survival of *E. coli*, and NaCl was used as a stress inducer for sucrose accumulation in *S. elongatus* UTEX 2973.

Before the two strains were cultivated together, *S. elongatus* UTEX 2973 was propagated in BG-11 at 37 °C with appropriate antibiotics to the exponential phase (OD_750_ ≈ 1.0), collected by centrifugation, inoculated into 25 mL of CoBG-11 medium and grown at 30 °C for 48 h to an OD_750_ of 0.5. *E. coli* was incubated in CoBG-11 with 1 g/L sucrose for 48 h and then collected by centrifugation, resuspended in deionized water and inoculated into the 25-mL *S. elongatus* culture described above at an initial OD_600_ of 0.01.

### Quantification of cyanobacteria and *E. coli*

For pure cultures of *S. elongatus* UTEX 2973 and *E. coli*, cell density was measured at OD_750_ and OD_600_, respectively, using a UV-1750 spectrophotometer (Shimadzu, Kyoto, Japan). For co-culture, serial dilutions were made, and solid LB agar plates were used to determine *E. coli* viability and cell number by counting colony-forming units (CFU) after 24 h of incubation at 37 °C. The cell number of *S. elongatus* UTEX 2973 was determined by a hemocytometer under a microscope (BX43, Olympus, Shinjuku, Tokyo, Japan).

### Determination of extracellular sucrose content

Supernatants of pure *S. elongatus* UTEX 2973 cultures were collected and analyzed for sucrose content via a colorimetric glucose–sucrose assay (Megazyme, Ireland) that employs high-purity glucose oxidase, peroxidase and β-fructosidase (invertase). At pH 4.6, sucrose is hydrolysed by invertase to d-glucose and d-fructose, and then, the free d-glucose content is determined by conversion to a red-colored quinoneimine dye compound through the action of glucose oxidase and peroxidase at pH 7.4 and employing *p*-hydroxybenzoic acid and 4-aminoantipyrine. Measurements were conducted at 510 nm.

### Quantification of 3-HP

The 3-HP concentration was quantified according to a previously described method [37]. A 3-HP standard of analytical purity was purchased from Tokyo Chemical Industry (Tokyo, Japan). The supernatant containing 3-HP was collected from the co-culture medium by centrifuging at 12,000 rpm for 2 min at room temperature (Eppendorf 5430R, Hamburg, Germany) and used for 3-HP analysis. Sample derivatization was carried out according to the two-stage technique described previously [76]. GC–MS analysis was conducted on a GC–MS system-GC 7890 coupled to an MSD 5975 (Agilent Technologies, Inc., Santa Clara, CA) equipped with an HP-5MS capillary column (30 mm × 250 mm id).

### Quantitative real-time RT-PCR analysis

Approximately 4 × 10^6^ pure or co-cultured *S. elongatus* UTEX 2973 cells were collected by centrifugation at 12,000 rpm and 4 °C for 1 min. The supernatant was removed, and the cells were used for RNA extraction and RT-qPCR analysis using methods described previously [77]. The relative abundance of different mRNA molecules could be estimated using 2^−ΔΔCT^ [78].

### Analysis of H_2_O_2_ concentration

The H_2_O_2_ content in the supernatant was analyzed using the H_2_O_2_ Quantitative Assay Kit (Sangon Biotech, Shanghai, China). In the reaction, Fe^2+^ is oxidized to Fe^3+^ by H_2_O_2_ when the pH is less than 7.0, and then, the generated Fe^3+^ combines with dye molecules to form a claret-colored Fe^3+^–dye complex with a maximum absorption wavelength of 560 nm or 595 nm, and the absorption value is directly proportional to the concentration of H_2_O_2_ in cells.

## Supplementary information

**Additional file 1: Fig. S1.** Growth of *E. coli* ABKm under different concentrations of NaCl in CoBG-11 medium at 37 °C.

**Additional file 2: Fig. S2.** Growth of *E. coli* cscN and ABKm. A), B), C) Cultivated in M9 medium; D), E), F) cultivated in CoBG-11 medium.

**Additional file 3: Fig. S3.** Growth in the artificial consortium system at 37 °C. *S. elongatus cscB*^+^ (green square) and *E. coli* ABKm (gray diamond).

**Additional file 4: Fig. S4.** Consumption of sucrose in the co-culture system.

**Additional file 5: Table S1.** Related genes and primers used in this study.

**Additional file 6: Fig. S5.** Growth of the cyanobacterium *S. elongatus cscB*^+^ with H_2_O_2_ added.

**Additional file 7: Fig. S6.** Expression level analysis of genes involved in photosynthesis. Gene expression analysis of *ccmM, rbcL, cp43, cp47, psaB, psaA. chlaA* and *pcrA* in *S. elongatus cscB*^+^. The error bars represent the calculated standard deviation of the measurements of three biological replicates.

## Data Availability

All data generated or analyzed during this study are included in this published article and its additional files.

## Abbreviations

GC–MS: Gas chromatography–mass spectrometry
WT: Wild type
3-HP: 3-Hydroxypropionic acid
F6P: Fructose 6-phosphate
PHA: Polyhydroxyalkanoates
PHB: Polyhydroxybutyrate
ROS: Reactive oxygen species
