# Supplementary figures and images for: Construction and analysis of an artificial consortium based on the fast-growing cyanobacterium Synechococcus elongatus UTEX 2973 to produce the platform chemical 3-hydroxypropionic acid from CO2
Source: Biotechnol Biofuels. 2020 May 6;13:82. doi: 10.1186/s13068-020-01720-0 (PMC7201998; doi:10.1186/s13068-020-01720-0)

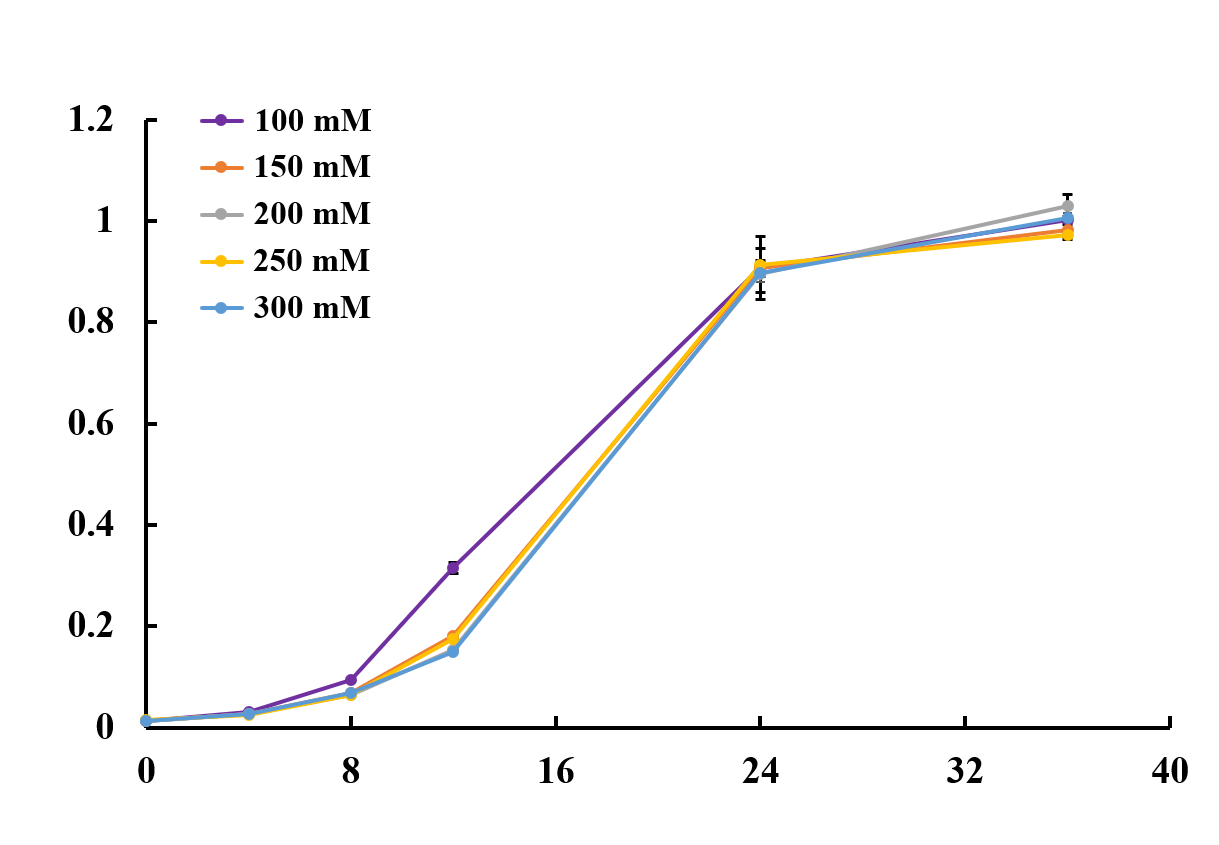

Supplement: Supplementary file 1 — Additional file 1: Fig. S1. Growth of E. coli ABKm under different concentrations of NaCl in CoBG-11 medium at 37 °C. [file 13068_2020_1720_MOESM1_ESM.tif]

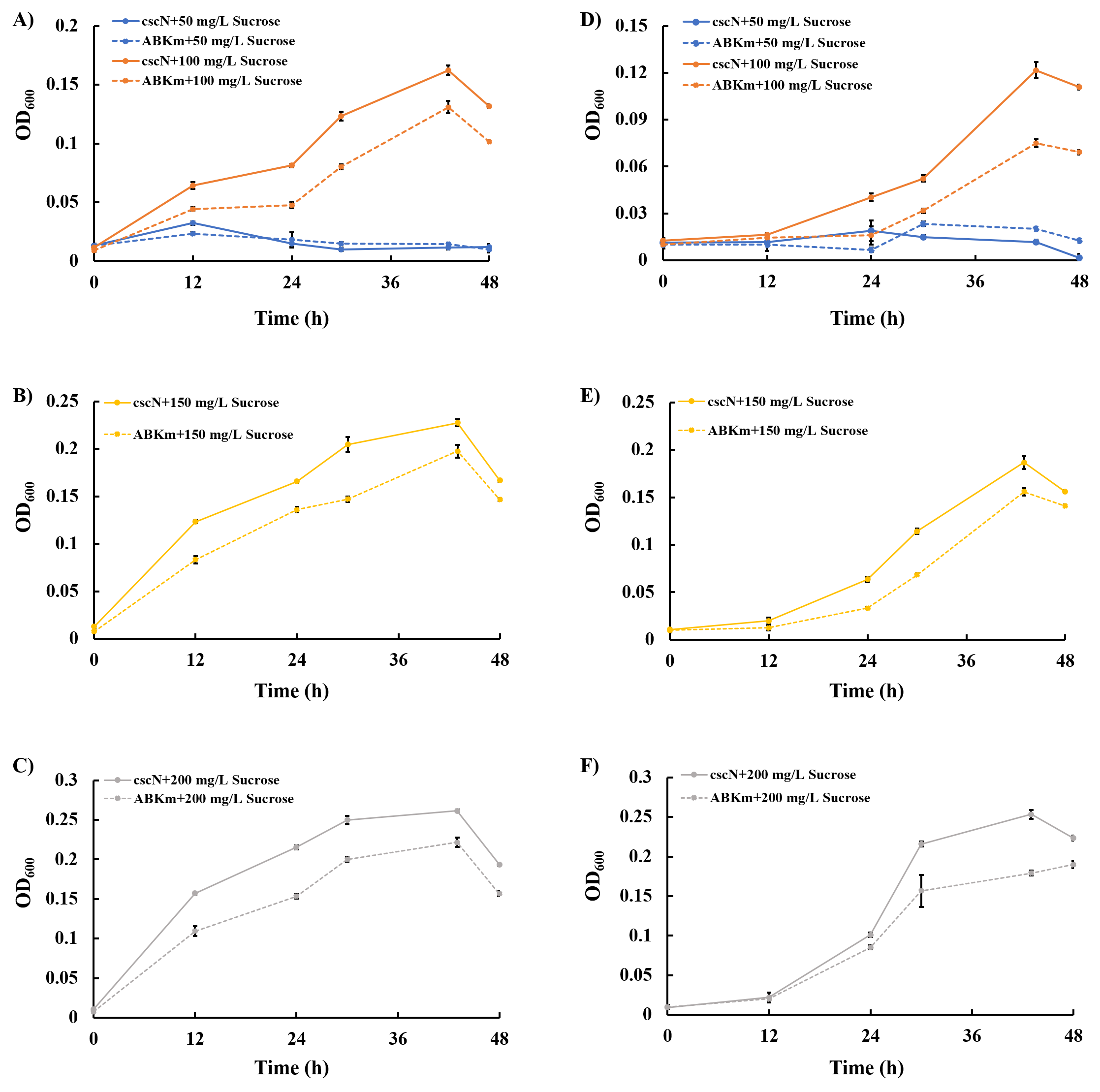

Supplement: Supplementary file 2 — Additional file 2: Fig. S2. Growth of E. coli cscN and ABKm. A), B), C) Cultivated in M9 medium; D), E), F) cultivated in CoBG-11 medium. [file 13068_2020_1720_MOESM2_ESM.tif]

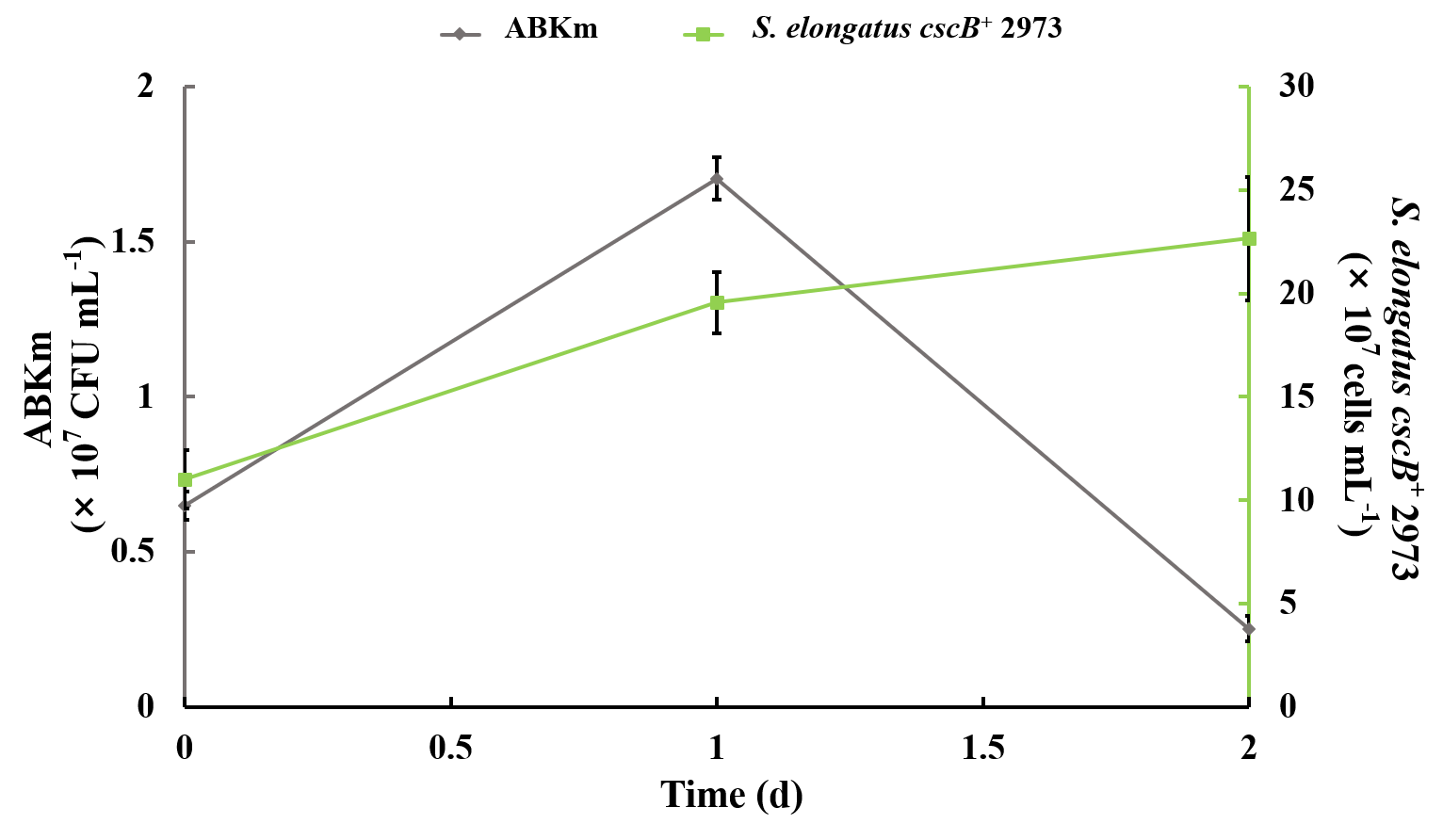

Supplement: Supplementary file 3 — Additional file 3: Fig. S3. Growth in the artificial consortium system at 37 °C. S. elongatus cscB+ (green square) and E. coli ABKm (gray diamond). [file 13068_2020_1720_MOESM3_ESM.tif]

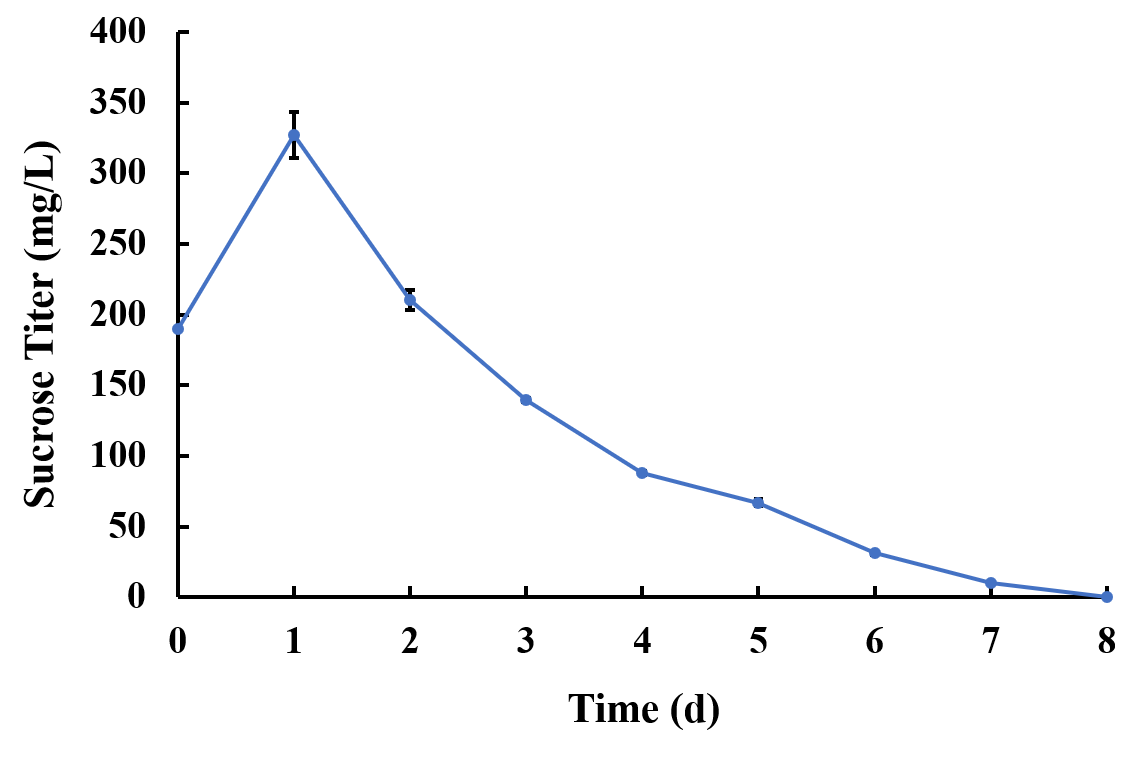

Supplement: Supplementary file 4 — Additional file 4: Fig. S4. Consumption of sucrose in the co-culture system. [file 13068_2020_1720_MOESM4_ESM.tif]

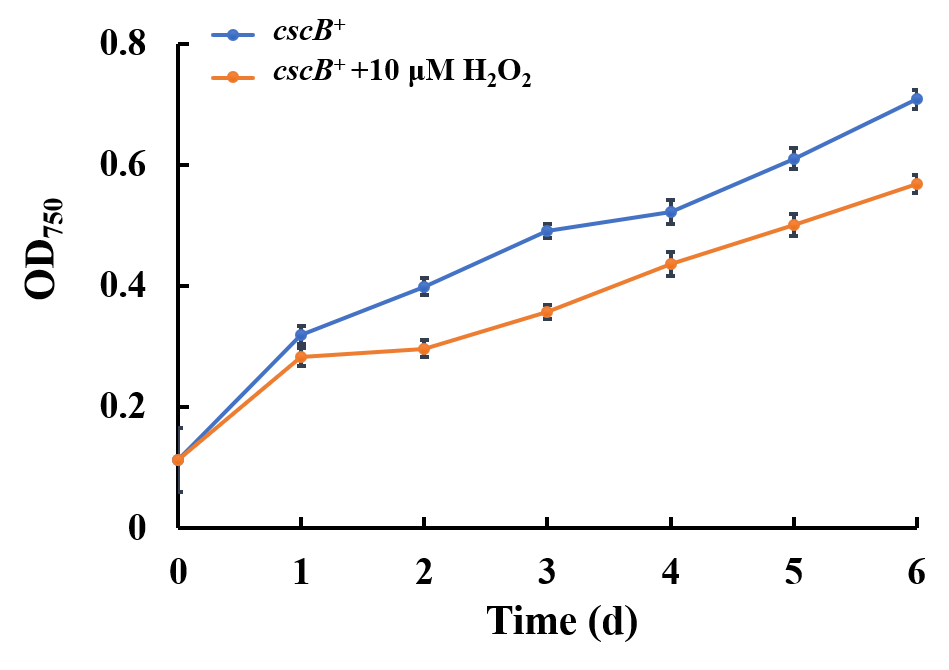

Supplement: Supplementary file 6 — Additional file 6: Fig. S5. Growth of the cyanobacterium S. elongatus cscB+ with H2O2 added. [file 13068_2020_1720_MOESM6_ESM.tif]

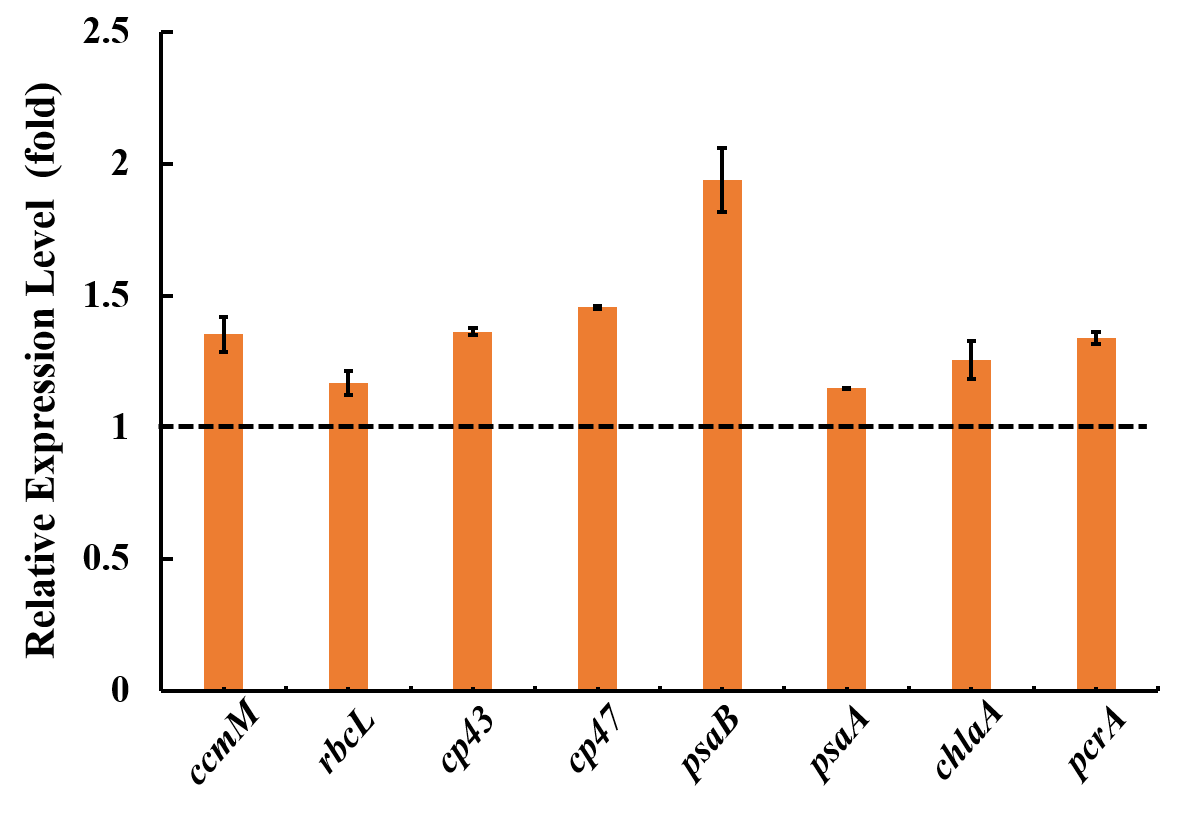

Supplement: Supplementary file 7 — Additional file 7: Fig. S6. Expression level analysis of genes involved in photosynthesis. Gene expression analysis of ccmM, rbcL, cp43, cp47, psaB, psaA. chlaA and pcrA in S. elongatus cscB+. The error bars represent the calculated standard deviation of the measurements of three biological replicates. [file 13068_2020_1720_MOESM7_ESM.tif]
