# Supplementary material for: Construction and analysis of an artificial consortium based on the fast-growing cyanobacterium Synechococcus elongatus UTEX 2973 to produce the platform chemical 3-hydroxypropionic acid from CO2
Source: Biotechnol Biofuels. 2020 May 6;13:82. doi: 10.1186/s13068-020-01720-0 (PMC7201998; doi:10.1186/s13068-020-01720-0)
Supplement: Supplementary file 5 — Additional file 5: Table S1. Related genes and primers used in this study. [file 13068_2020_1720_MOESM5_ESM.docx]

**Table S1. Related genes and primers used in this study.**

| **Genes** | |  | **Primers (5’→3’)** | |
| --- | --- | --- | --- | --- |
| 1 | *katG* (*ECD_03828*) | catalase-peroxidase HPI | F-AAAGCACAGCAACTGACGCT | R-ATTCGTCGGTCGCTTTCCAC |
| 2 | *katF* (*ECD_02591*) | catalase HPII | F-CGGAACGTGGTTTCCGCTTC | R-GCGATCTCTTCCGCACTTGG |
| 3 | *katE* (*ECD_01701*) | catalase HP | F-CCCACATCAGCACCAGTCAC | R-TGCCTTTGCGTACGTCTTCC |
| 4 | *sodB* (*M744_12745*) | superoxide dismutase | F-CCCTTTGACTACACGGCACT | R-GCATTGTTGAACAGACCGGC |
| 5 | *gpx1* (*M744_10575*) | glutathione peroxidase | F-GTCTGCAGGTCTTGGCGTTC | R-GTCAGCGCTGCGTAAAGAGG |
| 6 | *gpx2* (*M744_00775*) | glutathione peroxidase | F-CAGTCGTTTTCTCGACGGGCTA | R-GAGATCAACTGCAGCTTGGCG |
| 7 | *px* (*M744_06155*) | peroxidase | F-ACTCCCGTTTGCACGACTGA | R-TGACTTGGGTGCCTTGGGTT |
| 8 | *catP* (*M744_08205*) | peroxidase | F-GCACCAATCCCAACAGTCGC | R-CAGTTAGCGCCACCGTTTCC |
| 11 | *psbA* (*M744_00850*) | Photosystem II P680 reaction center D1 protein | F-GGCATGGAAGTGATGCACGA | R-AACCGTGAATTGAAGGCGCA |
| 12 | *ccmM* (*M744_09475*) | Carbon dioxide concentrating mechanism protein | F-CAGCCACAGCTACACCTCAA | R-GCAAGACAGTTGCTTCGCTC |
| 13 | *rbcL* (*M744_09460*) | Ribulose-bisphosphate carboxylase large chain | F-TATAAGGCCGGGGTGAAGGA | R-TCCAGGTACCGGTCGAAGAT |
| 14 | *cp43* (*M744_13530*) | Photosystem II CP43 chlorophyll apoprotein | F-CCCTGTCGTTGATGGGCTTC | R-GAGCTGAACCGATGTTGGCA |
| 15 | *cp47* (*M744_13305*) | Photosystem II CP47 chlorophyll apoprotein | F-CCACACGGTCGTCCTCAATG | R-GAACGGCAACACGAACATGC |
| 16 | *psaB* (*M744_06155*) | Photosystem I P700 chlorophyll a apoprotein A2 | F-CAGGATCCGACAACACGTCG | R-CTTGGCTCCACTGCTCGAAG |
| 17 | *psaA* (*M744_06150*) | Photosystem I P700 chlorophyll a apoprotein A1 | F-TACCGCACGAACTGGGGTAT | R-TGATCGAACCCAGGATGGCT |
| 18 | *chlaA* (*M744_05965*) | Chlorophyll a synthase | F-TCGCTTTTGGACTGGATCGC | R-GCGATGTAACTGGCACCCAA |
| 19 | *pcrA* (*M744_03720*) | Protochlorophyllide reductase | F-GCAGCAACCCACTGTCATCA | R-TCAGGCTGTAGTTCTCGGGG |
| 20 | *mcr* (*Caur_2614*) | Malonyl-CoA reductase | F- ATGAGCGGAACAGGACGACTGGCAG | R-TTACACGGTAATCGCCCGTCCGCG |
| 21 | *cscB* (*ECW_m2594*) | Sucrose permease | F- TTGTATGCCATCTGGTTGAAAG | R- CAAGACAAAATCCGCAAGGCCAT |
| 22 | *cscBKA* cluster | Sucrose metabolism | F- CCGGTTGAGGGATATAGAGCTATCGAC | R- CTGTTGATCCGTTGTTCCACCTGAT |
